# Hysteroscopic sutureless fixation of the levonorgestrel-releasing intrauterine system in the treatment of heavy menstrual bleeding

**DOI:** 10.3389/fmed.2025.1705266

**Published:** 2025-12-03

**Authors:** Linjiang Shen, Juan Zhang, Junjiang Wu, Mingming Ye, Hui Shao, Feng Zhang

**Affiliations:** Shaoxing Maternity and Child Health Care Hospital, Maternity and Child Health Care Affiliated Hospital, Shaoxing University, Zhejiang, China

**Keywords:** levonorgestrel-releasing intrauterine system (LNG-IUS), heavy menstrual bleeding (HMB), sutureless fixation, hysteroscope, suture fixation

## Abstract

**Background:**

This study investigated the safety and efficacy of hysteroscopic sutureless fixation of the levonorgestrel-releasing intrauterine system (LNG-IUS) for treating heavy menstrual bleeding (HMB), providing a viable therapeutic option for patients at high risk of LNG-IUS expulsion.

**Methods:**

Ninety patients were enrolled and equally divided into three groups: conventional implantation of LNG-IUS group (control group), hysteroscopic suture fixation of LNG-IUS group (suture fixation group) and hysteroscopic sutureless fixation of LNG-IUS group (sutureless fixation group). The rates of LNG-IUS expulsion and displacement, complication rates, pre- and postoperative menstrual flow, pain scores, and life satisfaction scores were recorded for each of the three groups.

**Results:**

The LNG-IUS expulsion rate in the sutureless fixation group was 3.3% and the displacement rate was 10%. There was no significant difference compared to the suture fixation group (3.3% LNG-IUS expulsion rate and 16.7% displacement rate in the suture fixation group, *p* > 0.05). There was a significant difference compared to the control group (13.3% expulsion rate and 23.3% displacement rate in the control group, *p* < 0.05). There was no significant difference in the rate of postoperative complications among the three groups (*p* > 0.05). Postoperative menstrual flow was significantly reduced in all three groups (*p* < 0.05), pain scores were significantly reduced (*p* < 0.05), and quality of life scores were significantly improved (*p* < 0.05).

**Conclusion:**

Our findings indicate that hysteroscopic sutureless fixation of the LNG-IUS is a safe, effective, and minimally invasive surgical procedure to prevent the LNG-IUS expulsion. This procedure leads to a significant reduction in menstrual flow, relieves the symptoms of dysmenorrhea, and improves patient quality of life.

## Background

Heavy menstrual bleeding (HMB), also known as menorrhagia, is characterized by a menstrual blood loss exceeding 80 mL (1). It is one of the most common reasons for gynecological consultations, affecting approximately 27.2–30% of women of reproductive age (2, 3). The levonorgestrel-releasing intrauterine system (LNG-IUS) is considered one of the most effective minimally invasive treatments for HMB and has been endorsed by numerous national guidelines (4).

However, underlying conditions such as adenomyosis and a larger uterine cavity can lead to a high risk of LNG-IUS expulsion. The expulsion rate in patients with adenomyosis has been reported to be approximately 25% (5). Other recognized risk factors for IUD expulsion include a history of previous expulsion, heavy menstrual bleeding or severe dysmenorrhea, postpartum insertion or after a second-trimester abortion, and multiple prior vaginal deliveries (6).

Several studies have explored hysteroscopic fixation techniques to mitigate expulsion. Zhang et al. (7) reported a new surgical protocol using a hysteroscopic cold knife system to suture the LNG-IUS to the myometrium, demonstrating promising results. Similarly, Peng et al. (5) reported an LNG-IUS expulsion rate of only 10.2% using a novel insertion technique. Despite these advances, current methods present certain limitations. This study aims to describe an innovative sutureless fixation technique for the LNG-IUS and to prospectively compare its efficacy and safety against hysteroscopic suture fixation and conventional implantation.

## Methods

With the approval of the Medical Ethics Committee of Shaoxing Maternal and Child Health Hospital, a total of 96 patients were recruited starting in January 2023. After accounting for loss to follow-up, 90 patients were ultimately included and divided into three groups: the normal hysteroscopic placement group (control group), the hysteroscopic suture fixation group, and the hysteroscopic sutureless fixation group. All participants provided written informed consent. Inclusion criteria were as follows: (a) presentation with symptoms of menorrhagia; (b) desire for uterine preservation; (c) non-pregnant status and no fertility plans in the short term; (d) no known allergy to the LNG-IUS; and (e) good compliance. Exclusion criteria included: (a) contraindications to progesterone therapy or hysteroscopy, such as severe coagulation disorders, severe cardiac, hepatic, or renal dysfunction, or mental disorders; (b) diagnosis of breast tumors; (c) history of female genital tuberculosis, submucous myoma, or endometrial carcinoma; and (d) postmenopausal status.

Treatment methods: Before treatment, a series of examinations were carried out on the patients, including routine gynecological examination, transvaginal ultrasonography, and basic general check-ups to exclude contraindications. The control group received conventional implantation of the LNG-IUS. Hysteroscopic suture fixation of LNG-IUS is based on the method of Zhang and Mao et al. (6, 7): Step 1 Join non-absorbable suture and the LNG-IUS together; Step 2 Insertion and suture fixation of the LNG-IUS; Step 3 Endoscopic knots; Step 4 Confirm the location of the LNG-IUS. All patients were successfully performed with hysteroscopic suture fixation of the LNG-IUS in the uterine cavity.

**Figure 1:**
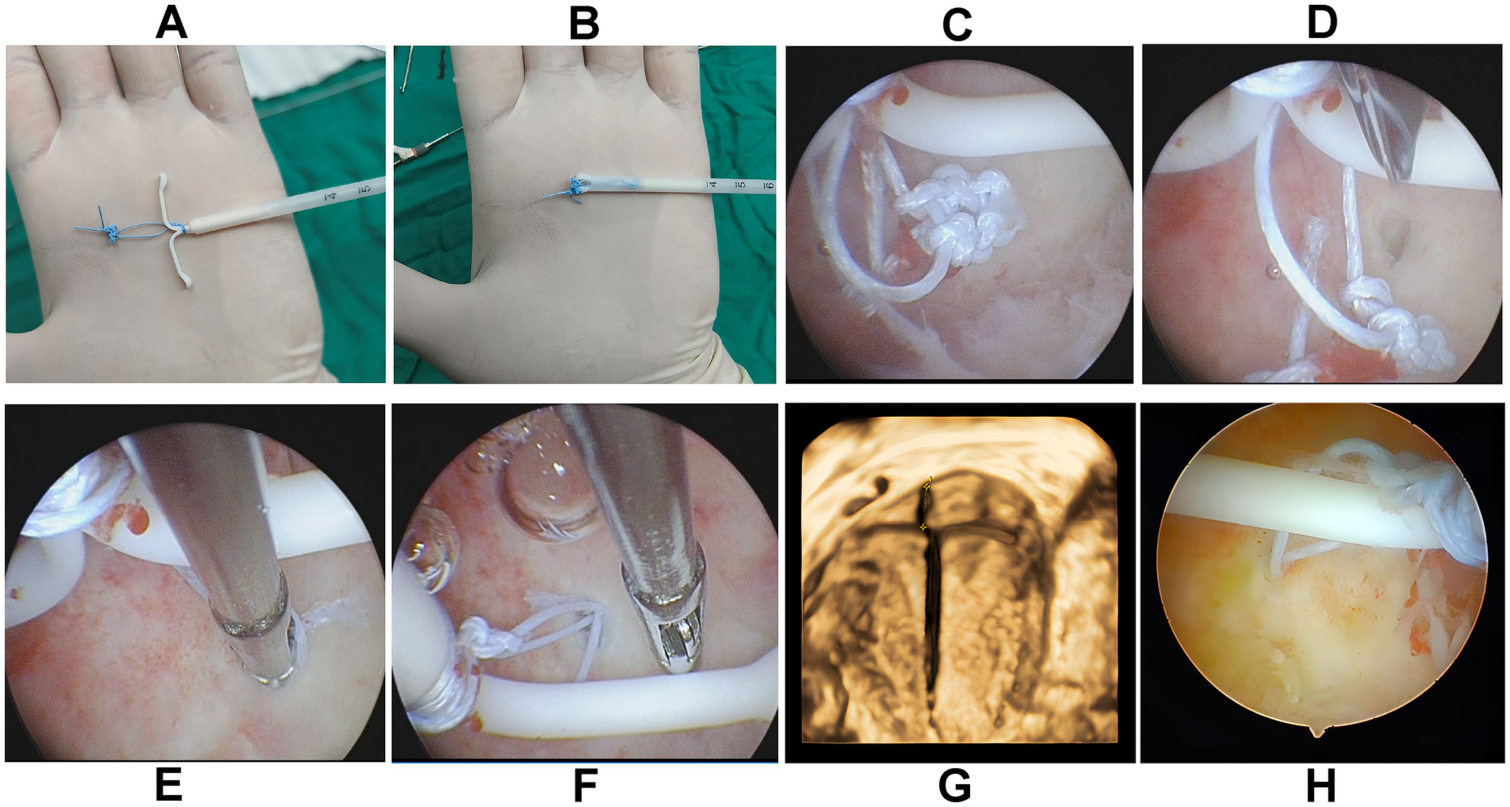
Hysteroscopic sutureless fixation of the LNG-IUS. **(A,B)** A non-absorbable wire knot is made at the upper end of the LNG-IUS, which is then retracted into the casing. **(C)** The LNG-IUS was inserted into the uterine cavity. **(D–F)** A small incision was made in the myometrium using scissors, and the knot head of the LNG-IUS was inserted into the myometrium using hysteroscopic forceps. **(G)** A three-dimensional ultrasound confirmed that the wire knot head was securely fixed within the myometrium of the uterine fundus. **(H)** A follow-up hysteroscopy examination after 1 month showed that the small incision in the myometrium had healed and the LNG-IUS had been properly fixed.

The surgical procedure for hysteroscopic sutureless fixation of a levonorgestrel-releasing intrauterine system (LNG-IUS), as illustrated in Figure 1, was performed as follows: A 2/0 non-absorbable suture was used to tie knots between the shank and the arms of the LNG-IUS, with an additional 6–8 knots created 0.8–1 cm away to form a knot head. The arms were then inserted into the insertion tube, enabling placement without contact with the vulva and vaginal wall to reduce contamination risk. The LNG-IUS was subsequently inserted into the uterine cavity in the conventional manner. Under ultrasound guidance, a small incision not exceeding 0.8 cm in depth was made in the myometrium using scissors to minimize the risk of uterine perforation. The knot head of the LNG-IUS was then inserted into the myometrial incision using hysteroscopic forceps. Finally, the hysteroscope was withdrawn, and the tail of the LNG-IUS filaments was cut off, thereby completing the procedure.

The patients were monitored at 1, 3, 6, and 12 months postoperatively through telephone interviews and outpatient visits. For each of the three groups, the rates of LNG-IUS expulsion and displacement, pre- and postoperative menstrual flow, complications, pain scores, and life satisfaction scores were documented. Menstrual blood loss was evaluated using the Pictorial Blood Loss Assessment Chart (PBAC) (8). Dysmenorrhea and treatment response were assessed with a Visual Analogue Scale (VAS) ranging from 0 to 10 (9). The Chinese version of the Euro Qol 5-Dimension (EQ-5D) scale was employed to measure health-related quality of life (HRQoL). The reliability and validity of the Chinese EQ-5D have been confirmed, and its equivalence to the English version has been established (10, 11). This instrument evaluates HRQoL across five dimensions: Mobility, Self-care, Usual Activities, Pain/Discomfort, and Anxiety/Depression. Each dimension is categorized into five severity levels: no problems (1), slight problems (2), moderate problems (3), severe problems (4), and unable/extreme problems (5). The resulting health states were converted into a single utility index score, where a higher value (on a scale where 1 represents full health) indicates a better quality of life.

### Statistical analysis

Continuous variables describing clinical characteristics are expressed as mean ± standard deviation and were compared between groups using the *t*-test. Categorical variables are presented as frequency (percentage) and were compared using the chi-square test or Fisher’s exact test, as appropriate. One-way analysis of variance (ANOVA) was used for comparing continuous variables across multiple groups. Data distribution and variability across groups were visually represented using boxplots. The box component (extending from the first to the third quartile) represents the interquartile range (IQR), covering the central 50% of the data. The vertical lines (whiskers) typically extend to the maximum and minimum values within 1.5*IQR, with outliers plotted individually. To enhance the robustness of the findings and quantify the magnitude of group differences, effect sizes were calculated and reported alongside *p*-values. A *p*-value < 0.05 was considered statistically significant. Statistical analyses were performed using SPSS version 21.0 and R version 4.4.2.

## Results

A total of 90 patients with heavy menstrual bleeding (HMB) completed the study, with 30 cases each allocated to the control group, the suture fixation group, and the sutureless fixation group. Baseline characteristics, including age, body mass index (BMI), parity, abortion history, and hemoglobin levels, were compared among the three groups. As shown in Table 1, no significant differences were observed in these parameters.

**Table 1:** Comparison of the clinical characteristics of patients before treatment.

Characteristics	Total (*n* = 90)	G1 (*n* = 30)	G2 (*n* = 30)	G3 (*n* = 30)	Statistic	*P*
Age, Mean ± SD	42.91 ± 5.18	42.70 ± 5.09	44.33 ± 5.35	41.70 ± 4.91	*F* = 2.02	0.139
BMI, Mean ± SD	24.25 ± 3.25	24.49 ± 2.15	23.43 ± 2.86	24.83 ± 4.30	*F* = 1.54	0.220
Number of abortions, Mean ± SD	1.61 ± 1.47	1.90 ± 1.86	1.77 ± 1.33	1.17 ± 1.02	*F* = 2.19	0.119
Number of births, Mean ± SD	1.41 ± 0.67	1.47 ± 0.90	1.40 ± 0.56	1.37 ± 0.49	*F* = 0.17	0.843
Hemoglobin level, Mean ± SD	107.24 ± 19.50	104.17 ± 18.58	105.27 ± 19.70	112.30 ± 19.82	*F* = 1.56	0.217

G1, suture fixation group; G2, sutureless fixation group; G3, control group; SD, standard deviation, F ANOVA, BMI, body mass index; LNG-IUS, levonorgestrel-releasing intrauterine system.

**Figure 2:**
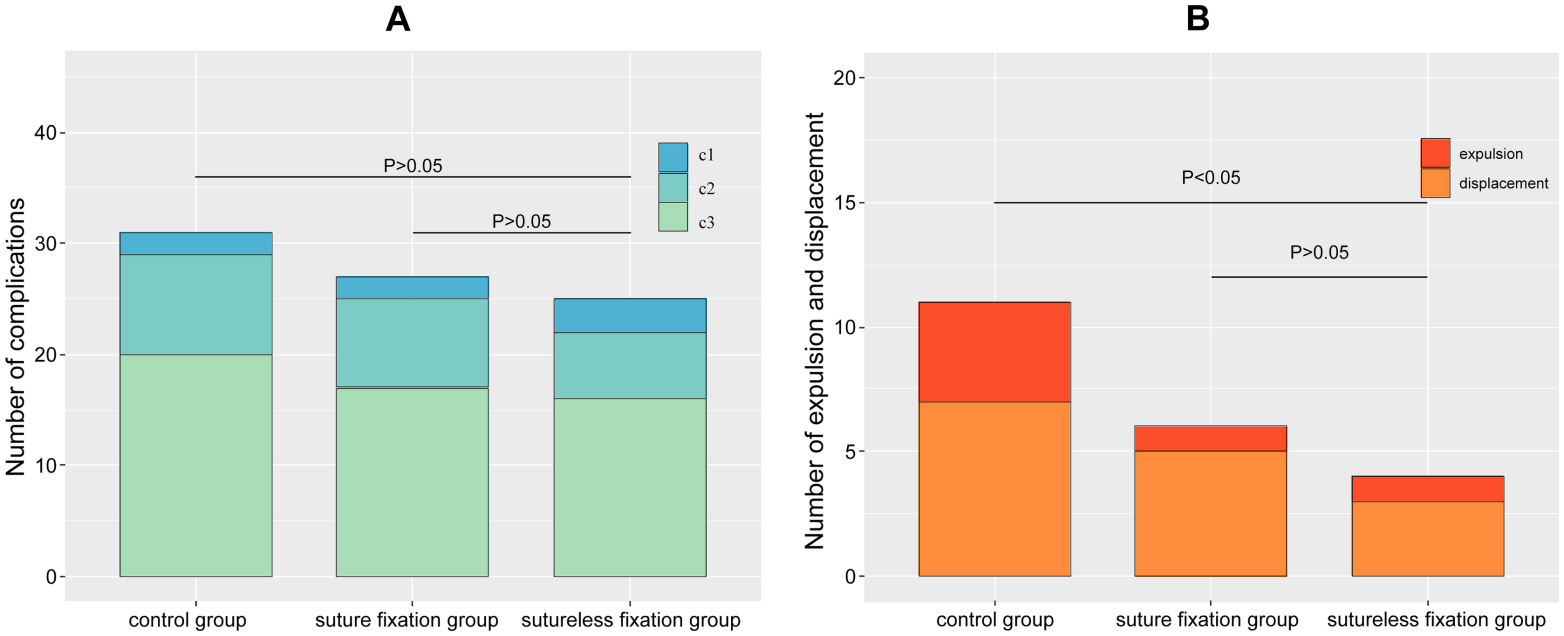
**(A)** Comparison of postoperative complications. There were no statistically significant differences in the above complications among the three groups. This demonstrates that both sutured and sutureless fixation of the LNG-IUS are safe and associated with minimal risks. **(B)** Comparison of exclusion and displacement rates in the three groups. There was no statistically significant difference in expulsion rates between the LNG-IUS suture fixation group and the sutureless fixation group. Both groups exhibited lower expulsion rates than the control group. This demonstrates that both LNG-IUS suture fixation and sutureless fixation effectively reduce expulsion rates. *c1* lower abdominal discomfort, *c2* yellow vaginal discharge, *c3* irregular vaginal bleeding.

No intraoperative complications—such as air embolism, intravascular absorption syndrome related to hysteroscopic surgery, infection, or uterine perforation—were observed in any of the three groups. In the sutureless fixation group, 16 patients experienced irregular vaginal bleeding, 6 had yellow vaginal discharge, and 3 reported symptoms such as lower abdominal discomfort postoperatively; however, these findings showed no statistically significant difference compared with the other two groups (*p* > 0.05), as presented in Figure 2A. The expulsion rate of LNG-IUS in the sutureless fixation group was 3.3%, and the displacement rate was 10%, both lower than the corresponding rates in the control group (13.3 and 23.3%, *p* < 0.05). No statistically significant difference was found when compared with the suture fixation group (3.3 and 16.7%, *p* > 0.05), as shown in Figure 2B.

**Figure 3:**
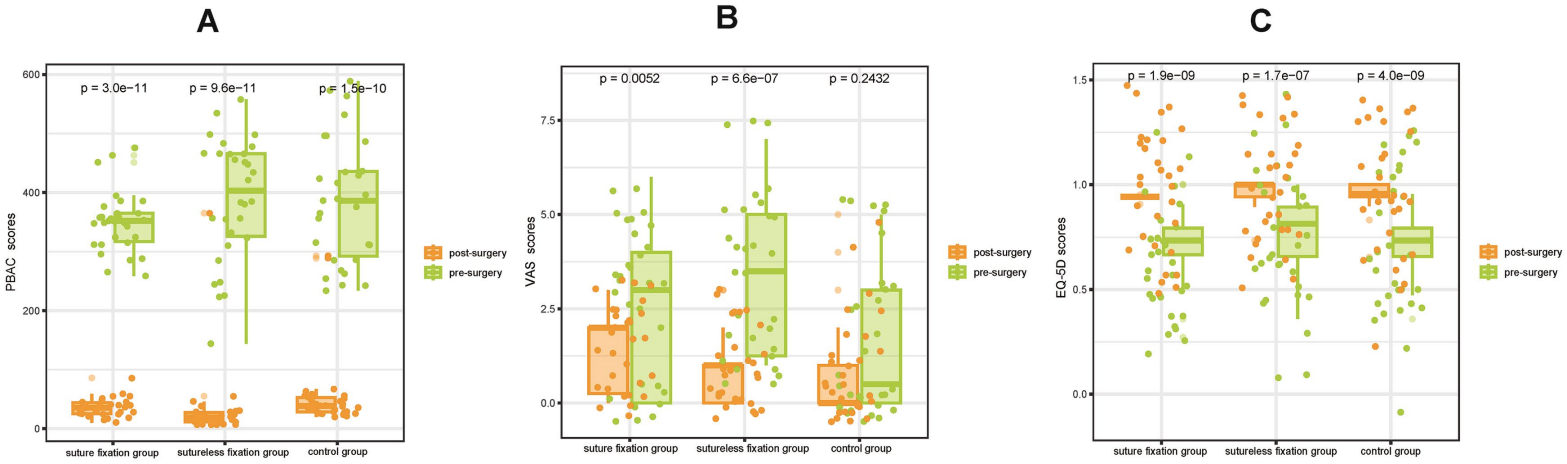
PBAC scores **(A)** and VAS scores **(B)** at 1 month postoperatively in all three groups showed significant decreases compared with preoperative scores, and EQ-5D scores **(C)** increased significantly compared with preoperative scores. Both the LNG-IUS suture fixation group and the sutureless fixation group effectively reduced menstrual flow, corrected anemia, alleviated pain, and improved patients’ quality of life. PBAC, pictorial blood loss assessment chart; VAS, visual analogue scale; EQ-5D, European quality of life five-dimension scale.

**Figure 4:**
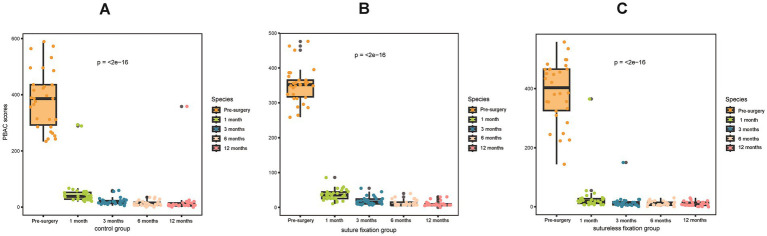
Comparison of preoperative and postoperative PBAC scores: postoperative PBAC scores decreased significantly in all three groups. This demonstrates that both LNG-IUS suture fixation and sutureless fixation are effective treatments for HMB. PBAC, pictorial blood loss assessment chart.

**Figure 5:**
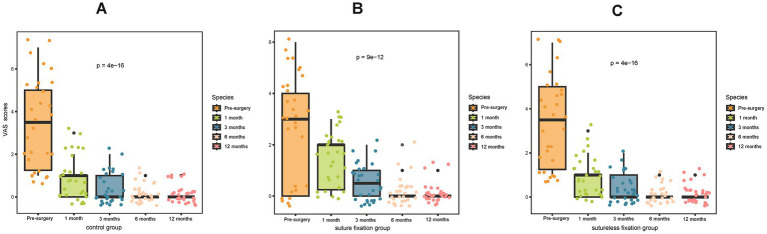
Comparison of preoperative and postoperative VAS scores: postoperative VAS scores decreased significantly in all three groups. This demonstrates that both the LNG-IUS suture fixation and sutureless fixation can effectively alleviate dysmenorrhea symptoms. VAS, visual analogue scale.

In the first month after surgery, all three groups demonstrated significant improvements in menstrual flow and dysmenorrhea, as reflected by markedly reduced PBAC and VAS scores (*p* < 0.05; Figures 3A,B). Life satisfaction also increased significantly in all groups, with notably higher EQ-5D scores compared to preoperative values (*p* < 0.05; Figure 3C). Throughout the 12-month follow-up period, both menstrual flow and dysmenorrhea continued to improve significantly relative to the preoperative state, accompanied by further decreases in PBAC and VAS scores (*p* < 0.05; Figures 4, 5). By 1 year post-surgery, none of the patients in any group presented with anemia, and their EQ-5D scores had reached full or near-full levels.

## Discussion

According to the UK National Institute for Health and Care Excellence (NICE) guidelines, heavy menstrual bleeding (HMB) is defined as excessive menstrual blood loss that interferes with a woman’s physical, social, emotional, and/or material quality of life. It can occur alone or in combination with other symptoms. HMB is highly prevalent among women, with a large European epidemiological survey reporting a prevalence of 27.2% in women aged 18–57 years (3), and approximately one-third of perimenopausal women experiencing HMB (12). The etiology of HMB is diverse and includes uterine fibroids, adenomyosis, ovulatory disorders, systemic coagulopathies, and local endometrial abnormalities.

Currently, there are four primary treatment approaches for heavy menstrual bleeding: hysterectomy, endometrial resection or ablation, the levonorgestrel-releasing intrauterine system (LNG-IUS), and oral medication. Oral medications typically reduce HMB by approximately 20 to 45% (4). The LNG-IUS is a highly effective option for decreasing menstrual blood loss and is recommended in multiple clinical guidelines. According to the 2018 NICE guidelines, LNG-IUS is recommended as a first-line treatment for HMB associated with uterine fibroids smaller than 3 cm in diameter without uterine cavity distortion, as well as for suspected or diagnosed adenomyosis (13). Furthermore, the 2013 American College of Obstetricians and Gynecologists (ACOG) guidelines state that the LNG-IUS is effective in managing abnormal uterine bleeding (AUB), can reduce menstrual blood loss, and should be considered for patients across all age groups (14).

The advantages of LNG-IUS in treating HMB are as follows: Unlike conventional oral medications, which achieve an efficacy rate of 20–45%, the LNG-IUS can reduce menstrual bleeding in all women, irrespective of their prior menstrual bleeding patterns, and significantly improve their quality of life. The LNG-IUS demonstrates efficacy comparable to first-generation transcervical endometrial resection and second-generation hot-balloon endometrial ablation—both of which also markedly reduce menstrual bleeding and enhance quality of life. However, unlike endometrial resection or ablation, which are irreversible procedures, the LNG-IUS is less costly and can be employed as maintenance therapy following endometrial ablation to lower the risk of postoperative recurrence (15). Moreover, compared to hysterectomy, the LNG-IUS preserves fertility, is reversible, more cost-effective, and carries fewer risks, rendering it a more acceptable option for patients.

However, the primary risk associated with LNG-IUS treatment for menorrhagia is expulsion. Literature reports indicate expulsion rates ranging from 4.9 to 7.7% in the general population. In patients with adenomyosis, the cumulative expulsion rate over 12 months may reach 11%, attributed to uterine enlargement and heavy menstrual bleeding (16). Our study demonstrated that among HMB patients receiving conventional LNG-IUS placement, 13.3% experienced expulsion and 23.3% experienced displacement within 1 year, outcomes that adversely affect both patient health and economic outcomes.

Over the past years, an innovative technique involving fixation of the LNG-IUS to the myometrium using non-absorbable sutures has been reported in the literature as a solution to this issue, with promising outcomes (6, 7). Our institution began offering this procedure to suitable patients in 2022. However, several practical challenges have been observed: the suture fixation technique presents a steep learning curve, requiring systematic training and substantial practice for proficiency, typically necessitating an assistant; the procedure is only feasible in relatively large uterine cavities, with our experience showing frequent failures in cavities under 8 cm depth; moreover, it necessitates an additional hysteroscopy for LNG-IUS removal after 5 years, creating patient inconvenience and financial burden, while forced removal by an uninformed clinician could cause myometrial tearing and hemorrhage with potential serious complications.

To address the aforementioned challenges, we have developed an improved fixation method for the LNG-IUS. Our sutureless fixation technique offers several key advantages: 1. Simplicity and Efficiency: The procedure is straightforward and requires no specialized training, enabling even junior physicians to perform it independently without assistance. This reduces the demand for medical personnel, shortens operative time, and lowers surgical risks. 2. Broad Applicability: This method is suitable for any patient at high risk of LNG-IUS expulsion, including those with HMB, adenomyosis, or a previous history of IUD expulsion or displacement. It can be applied to uteri of all types and sizes, including those with anatomical anomalies. In contrast, sutured fixation is only feasible in relatively large uteri, as normal or small uterine cavities make suturing technically challenging, increase the likelihood of surgical failure, and may lead to inadequate fixation that raises the risk of postoperative expulsion. 3. Ease of Removal: Unlike sutured fixation, the sutureless technique does not require hysteroscopic assistance for removal after 5 years. The device can be retrieved by simply pulling the tail string, following standard removal procedures. Moreover, while patients with sutured LNG-IUS are vulnerable to severe bleeding or myometrial tearing if removal is attempted by a surgeon unfamiliar with the original technique—especially if the sutures are not hysteroscopically released—this risk is entirely avoided with the sutureless approach.

Previous studies have also employed different methods to secure the LNG-IUS. Peng FS et al. employed a novel technique (Yang’s method) (5) to secure the LNG-IUS, reducing its expulsion rate from 25.3 to 10.2%. However, unlike our suture-based fixation and sutureless fixation methods, this procedure is not performed under direct visualization and cannot achieve highly precise securing of the LNG-IUS. Second, the expulsion rate of the LNG-IUS using this method differs significantly from our approach, where both suture fixation and suture-free fixation methods achieved an expulsion rate of 3.3%. Consequently, this method has not been widely adopted to date. In contrast, our technique offers distinct advantages for widespread implementation: 1. True direct visualization enables highly precise fixation of the LNG-IUS; 2. Simple and easy operability; 3. Lower expulsion rate. We are confident that our technique will gain widespread adoption and promotion in the coming years.

Compared to routine LNG-IUS insertion, this technique involves additional hysteroscopic surgery and anesthesia, leading to higher upfront costs than standard outpatient placement. Its key clinical advantage, however, lies in significantly reducing the expulsion rate in high-risk patients—from an estimated 20–30% to below 5%. By minimizing expulsions, the procedure also lowers the need for secondary interventions and IUS replacements. Each prevented expulsion avoids the full cost of a repeat procedure, representing the most substantial source of cost savings. Additional savings come from reduced emergency visits, imaging studies, medications, and complications associated with malposition. Therefore, although the initial investment is higher, over the medium to long term, the cumulative costs for patients receiving routine insertions—who may require multiple replacements—can quickly exceed those of the sutureless fixation group. The “one-time success” of this approach avoids recurrent expenditures, ultimately lowering total healthcare costs. This strategy exemplifies a “front-loaded investment, long-term return” model. By shifting resources from managing repeated failures to ensuring treatment success in a single procedure, it improves clinical outcomes and patient quality of life while generating systemic cost savings. We therefore recommend prioritizing this technique in high-risk populations.

Our study demonstrated that following fixation (irrespective of the fixation technique used), the rates of LNG-IUS expulsion and displacement were significantly reduced without compromising postoperative outcomes, while substantially improving patients’ quality of life. Follow-up results indicated only one case of expulsion in the sutureless fixation group, likely attributable to an undersized fixation knot or insufficient embedding depth within the fundal myometrium. Nevertheless, this study has several limitations: 1. Additional data are required to further validate the safety and efficacy of the sutureless fixation approach; 2. Longer follow-up is necessary to assess the safety of non-hysteroscopic removal after 5 years of use; 3. The potential impact of the residual fundal myometrial micro-perforation following LNG-IUS removal on future pregnancy and delivery outcomes remains unclear. All these aspects warrant further investigation and extended follow-up in future studies.

## Conclusion

Hysteroscopic sutureless fixation of the LNG-IUS is a viable therapeutic strategy for women suffering from heavy menstrual bleeding (HMB) and those at high risk of LNG-IUS expulsion. This minimally invasive procedure not only effectively reduces menstrual blood loss and minimizes expulsion rates but also enhances overall patient quality of life.

## Data Availability

The raw data supporting the conclusions of this article will be made available by the authors, without undue reservation.
